# Bridging the Gap between Brain Activity and Cognition: Beyond the Different Tales of fMRI Data Analysis

**DOI:** 10.3389/fnins.2017.00031

**Published:** 2017-01-31

**Authors:** Maria G. Di Bono, Konstantinos Priftis, Carlo Umiltà

**Affiliations:** Department of General Psychology, University of PadovaPadova, Italy

**Keywords:** fMRI, multi-voxel pattern analysis, functional connectivity, effective connectivity, complex networks, graph-theoretical analysis, deep networks

The human brain is an extremely complex system of interacting physical and functional units, ranging from single neurons to complex networks. Cognition is a network phenomenon because it does not exist in isolated synapses, neurons, or even brain areas. In spite of that, a great amount of functional magnetic resonance imaging (fMRI) studies have explored what areas are involved in a variety of cognitive processes, merely localizing where in the brain those processes occur. Instead, the very notion of network phenomena requires understanding spatiotemporal dynamics, which, in turn, depends on the way fMRI data are analyzed. What are the mechanisms for simulating different cognitive functions and their spatiotemporal activity patterns? In order to bridge the gap between brain network activity and the emerging cognitive functions, we need more plausible computational models, which should reflect putative neural mechanisms and the properties of brain network dynamics.

## The Tales

With the advent of fMRI, neuroscientists have focused on the neuroanatomical localization of stimulus/task-induced changes in the blood-oxygenation level dependent (BOLD) signal. Indeed, analysis of fMRI data has been mainly based on univariate methods (i.e., the General Linear Model—GLM), which impose a series of critical assumptions and constraints. Crucially, the GLM is a voxel-wise analysis, in which each voxel time-series is analyzed independently, ignoring functional interactions among voxels within adjacent or non-adjacent brain areas. In addition, the GLM assumes a predefined shape of the Hemodynamic Response Function (HRF), which is convolved with each stimulus or task event for creating a hypothetical model of brain activity. Subsequently, multiple linear regression is used to search for voxels correlated with the predicted response. The HRF, however, may differ from the a priori assumed shape (Aguirre et al., 1998; Handwerker et al., 2004). Another critical point is the systematic use of spatial smoothing in the pre-processing phase. Spatial smoothing can dramatically increase the probability of false positives (Stelzer et al., 2014) and might cancel out differences between anatomically adjacent, but functionally distinct, brain areas. Hence, many aspects of the GLM were severely criticized (e.g., O'Toole et al., 2007; Stelzer et al., 2014).

In recent years, the Multivoxel Pattern Analysis (MVPA) has been extensively employed for analysing fMRI data. MVPA has done away with the GLM assumptions because it is a multivariate approach, for which neither spatial smoothing nor a parametric model of the HRF is required. Typically, a classifier is trained to distinguish trials among different conditions, using information coded within patterns of voxel activity. The trained model is then tested, through a cross-validation procedure, by predicting the conditions of the remaining (independent) data (Pereira et al., 2009). Classifiers were largely employed for predicting specific cognitive states in perceptual (e.g., Haynes and Rees, 2005; Kamitani and Tong, 2005, 2006) and other domains, like numerical cognition and motor control (e.g., Di Bono and Zorzi, 2008; Eger et al., 2009; Gallivan et al., 2011; Zorzi et al., 2011; Di Bono et al., 2015). MVPA can also capture temporal dynamics of brain networks, when used on spatiotemporal patterns of brain activity. Being able to predict cognitive states with a classifier, however, does not mean that we have understood what kind of spatial/spatiotemporal representation is encoded by brain activity. How can we break these codes? Representational similarity analysis (RSA) by Kriegeskorte et al. (2008) partially answers this question: for each region of interest, a similarity metric (e.g., correlation) is computed between pairs of distributed activity patterns representing different experimental conditions. In the same vein, multivariate cross-classification has been used for characterizing abstraction in neural representations across cognitive domains (for review, see Kaplan et al., 2015).

In addition, functional connectivity (FC) analysis can capture brain dynamics. FC allows one to identify significant brain networks with a coherent activity, either while a task is being performed or during a resting state. Indeed, by identifying changes in neuronal activity that are significantly predicted by stimulus manipulation, we see only part of the story. In effect, there is another part of brain activity that is internally generated. It must be kept in mind that the brain is continuously active, even in the absence of stimulation, and, therefore, quantifying stimulus-response relations alone does not fully capture brain dynamics. That is because stimulus-response relations might well be influenced by such “spontaneous” activity. Resting-state network analysis has increased our understanding of brain functional organization. FC analysis of resting-state fMRI (rs-fMRI) data has proved to be a powerful tool for investigating brain functional organization, both in healthy people and in patients (e.g., Baldassarre et al., 2014; Bassett and Bullmore, 2009). Traditional methods for analyzing FC in resting state mostly rested on a seed-based approach (Cole et al., 2010). Multivariate data-driven methods, like independent component analysis (ICA), principal component analysis (PCA), or clustering procedures (e.g., k-means, fuzzy c-means) offer an alternative way for identifying spontaneous coherent brain activity (McIntosh and Lobaugh, 2004; Beckmann et al., 2005, 2009; Lee et al., 2012).

The intrinsic limit of FC, however, is that its results are correlational in nature and, as such, do not index causality. If two regions are temporally correlated, there is no way of knowing whether one region influences the other (i.e., causality), or rather a third region affects both (i.e., mere correlation).

Effective-connectivity (EC) analysis can tackle this question. EC has been used to explore the possible causal influence of the activity in certain brain regions on the activity of other brain regions. Classic approaches for analyzing EC are based on Granger Causality (GC—Friston, 1994; Büchel and Friston, 2000), which captures only linear interactions. The dynamic causal modeling (DCM) of Friston et al. (2003) captures non-linear interactions (Friston et al., 2003; Stephan et al., 2008), but requires knowledge about the input to the system, as well as a priori knowledge about connectivity of the investigated network (Friston et al., 2003). DCM compares evidence for several competing a priori models with respect to the observed data (Penny et al., 2004). It may not be optimal for exploratory analyses (e.g., for studying resting state), although a new version of the DCM for resting state analysis has been proposed (Friston et al., 2014). A critical limit of DCM is that model selection procedures for connectivity should include more than just a few brain structures (for a critical review, see Roebroeck et al., 2011). Information theory also provides an excellent basis for formulating causal hypotheses, especially in the case of exploratory analyses. For example, Transfer Entropy (Schreiber, 2000) is a model-free measure, which is able to capture linear and non-linear causal interactions (e.g., Vicente et al., 2011). The preservation of temporal dependencies is mandatory when investigating causality because causes have to precede their effects. However, the temporal precedence might exist only at a certain time scale (e.g., milliseconds), and it is a potentially confounding concept when analysing fMRI time series, because of the regional variability of hemodynamic properties (David et al., 2008).

The analysis of FC and EC on rs-fMRI data (as described above) cannot describe both segregation and integration properties of brain functioning. Instead, graph-theoretical analysis provides a mathematical language for describing these properties, allowing one to analyze functional interactions among brain voxels at a topological level (Bullmore and Sporns, 2009; Sporns, 2011). The brain is modeled as a graph in which each node (e.g., each brain area) is linked to all the other nodes within the graph, through edges that are weighted by some measure of linear or non-linear functional correlation (or by some measure of EC). Numerous mathematical measures characterize graph topology, both at the global level of the graph structure and at the local level of constituent nodes (for details, see Rubinov and Sporns, 2010). Graph metrics provide evidence of both segregation (e.g., modularity and clustering) and integration (e.g., efficiency) properties of the graph. An emergent property of many complex networks is the “small-world” topology (Watts and Strogatz, 1998), which is in-between regular (i.e., each node is linked only to its neighbors) and random (i.e., each node is randomly connected to all the other nodes) graph topologies. Small-worldness characterizes graphs with dense local clustering and relatively few long-range connections, which is an appealing property, because it can globally account for both specialized (segregated) and distributed (integrated) information processing. In order to compute small-worldness, the standard quantitative application is to compare path length (a measure of distributed processing) and clustering (a measure of regional specialization), to an equivalent random network. It is interesting to note, however, that the small-world property seems to be less ubiquitous than suggested in the current literature. Telesford et al. (2011) have proposed a new small-world metric (ω) that compares network clustering to an equivalent lattice network, and path length to a random network. The ω metric accurately identifies small-world networks. Critically, the authors showed examples of networks that would be interpreted as small-world when the clustering coefficient is compared to a random network, but are not small-world according to ω. This is just an example of the critical points (including all the mathematical procedures needed to define the final network metrics) to be carefully considered when using graph theory in network neuroscience.

The investigation of how these topological properties are modulated by experimental manipulations has allowed neuroscientists to move from the level of representational codes to a level (still merely descriptive, though) of the mechanisms mediating the transition among different representations.

Indeed, understanding brain functioning is not only a matter of localizing functions and/or representations. Rather, we need to understand what are the mechanisms driving the transformation of such representations during different cognitive processes. We believe that graph theory is an excellent framework for topologically describing these mechanisms. The challenging question is: what is the learning mechanism, which, within spatial/anatomical constraints, has shaped the flexible representational code of the brain? Can we simulate it in a realistic way?

## Beyond the Tales

Conceiving the brain as a complex network has been the prevalent view in connectionist models, deriving from the principles of parallel and distributed information processing (PDP; McClelland et al., 1986). These models are intrinsically linked to the temporal dynamics of undirected/directed graphs, and their learning mechanism(s) should help us understand how cognition emerges from the activity of a complex network. In the latest generation of PDP models, hierarchical generative models, like Deep Belief Networks (Hinton, 2007), have been the main focus of interest in computational modeling. The reason for the interest in hierarchical generative models is attributable to their biological plausibility in terms of auto-organization, hierarchy, and unsupervised learning capability in a probabilistic fashion. These models are structured into a hierarchical composition of complete bipartite graphs (i.e., Restricted Boltzman Machines; Hinton and Salakhutdinov, 2006), and learn to reconstruct their input by discovering latent structures of the sensory data. In these networks, the analysis of the internal representations, both in terms of single-neuron activity (e.g., De Filippo De Grazia et al., 2012; Stoianov and Zorzi, 2012; Di Bono and Zorzi, 2013) and layer-pattern activity (e.g., Di Bono and Zorzi, 2013), has revealed emergent coding strategies, which closely mirror single-cell recording and neuroimaging data. Nonetheless, because only between- but no within-layer bidirectional connections are present, the biological plausibility of these models needs to be improved.

In our view, time is ripe for neuroimaging data to converge into the computational modeling ground, and for us to understand what kind of complex network/graphical model is the brain. We believe that graph theory can help us to construct a consistent empirical network model of the brain across the life span. Also, we believe that hierarchical generative models are a promising framework for constructing a more realistic brain network model. New plausible computational models are needed, which explain how complex brain networks can emerge and evolve mirroring biological complex systems. We have to understand what are the more plausible and efficient learning mechanisms, which, under physical/structural constraints, can allow the emergence of topological properties of segregation and integration within the brain, such as small-worldness, modularity, and rich-club organization. Because representing connectivity as a graph definitely enables the application of the same inference methods, across modalities, scales and experimental paradigms, graph theory provides a common language for better describing and understanding non-linear representations within computational network architectures. This is a yet unexplored area in computational modeling. We do not know whether functional dynamics within hierarchical generative models are topologically organized according to the same principles as those of complex brain networks. Finally, we expect that virtual “lesions” to those computational models provide evidence concerning the topology modulation, in accordance with neuropsychological findings.

## Conclusion

The human brain is a complex, dynamic-adaptive system of networks, from which cognition emerges. This viewpoint has led to a new era for neuroimaging, where graph theory is an excellent framework for topologically describing the mechanisms underlying cognition. We believe that time is ripe for neuroimaging to converge into the common ground of computational models, where hierarchical generative models represent a promising starting point for explaining these mechanisms in a probabilistic fashion.

## Author contributions

MGDB conceived the main idea. CU, KP equally contributed to the discussion of ideas. MGDB wrote the manuscript. KP, CU critically revised the manuscript.

### Conflict of interest statement

The authors declare that the research was conducted in the absence of any commercial or financial relationships that could be construed as a potential conflict of interest.
